# Discriminative ability of quality of life measures in multiple sclerosis

**DOI:** 10.1186/s12955-017-0828-0

**Published:** 2017-12-21

**Authors:** Kirsten M. Fiest, Jamie Greenfield, Luanne M. Metz, Scott B. Patten, Nathalie Jetté, Ruth Ann Marrie

**Affiliations:** 1 Department of Internal Medicine, University of Manitoba, GF533, 820 Sherbrook Street, Winnipeg, MB R3A 1R9 Canada; 2 Department of Clinical Neurosciences, University of Calgary, Calgary, AB Canada; 3 Hotchkiss Brain Institute, University of Calgary, Calgary, AB Canada; 4 Department of Community Health Sciences, University of Calgary, Calgary, AB Canada; 5 Department of Psychiatry, University of Calgary, Calgary, AB Canada; 6 Mathison Centre for Mental Health Research and Education, University of Calgary, Calgary, AB Canada; 7 O’Brien Institute for Public Health, University of Calgary, Calgary, AB Canada; 8 Department of Community Health Sciences, University of Manitoba, Winnipeg, MB Canada

**Keywords:** Multiple sclerosis, Quality of life, Health utility, Comorbidity, Concordance

## Abstract

**Background:**

Though many people with multiple sclerosis (MS) have comorbidities, the use of generic and disease-specific health related quality of life (HRQOL) scales to discriminate the effects of comorbidity has not been established. The utility of these scales to discriminate differences between persons with varying levels of disability is also unknown.

**Methods:**

Using online questionnaires, a convenience sample of Albertans with MS was recruited between July 2011 and March 2013. Participants completed demographic questions, a validated comorbidity questionnaire, a self-reported disability scale, and the following HRQOL scales: the Short Form (SF)-36, SF-6D, Health Utilities Index-Mark III (HUI-III), and Multiple Sclerosis Quality of Life-54 (MSQOL-54). The ability of each HRQOL scale to distinguish between comorbidity groups was assessed using a one-way analysis of covariance, adjusting for age, sex, disease course, and disability level.

**Results:**

Five hundred sixty three participants completed all relevant questionnaires. All HRQOL measures distinguished between persons with or without depression, while none were able to distinguish between participants with or without hypertension, thyroid disease, irritable bowel syndrome, or osteoporosis. The SF-36 physical scale, SF-6D, HUI-III, and MSQOL-54 physical scales were able to distinguish between all disability groups, though the HUI-III was better able to distinguish between individuals with moderate versus severe disability.

**Conclusions:**

Disease-specific measures would discriminate better between those with and without comorbidities than generic-specific measures and the HUI-III would discriminate best between persons with differing severities of disability. Generic or disease-specific measures may be useful in future studies examining the effects of comorbidity in MS and the effects of treatment of comorbidities in MS.

**Electronic supplementary material:**

The online version of this article (10.1186/s12955-017-0828-0) contains supplementary material, which is available to authorized users.

## Background

Comorbidity is increasingly recognized to be common in multiple sclerosis (MS), including physical and psychiatric comorbidity [1]; over 40% of the MS population may suffer from comorbidities depending on the age and sex of the population [2]. Some studies suggest that the presence of certain comorbidities is associated with delayed diagnosis, accelerated disability progression, and an increased risk of gadolinium-enhancing lesions on MRI [3, 4].

Several studies also report that physical and psychiatric comorbidity adversely affect health-related quality of life (HRQOL), suggesting that improved treatment of comorbidity may be one means of improving HRQOL in MS [5–7]. However, prior studies of the association between comorbidity and HRQOL differ with respect to the instruments used to assess HRQOL [8–11]. Instruments used in prior comorbidity studies in MS include the Short Form (SF)-12 [12], SF-36 [9], the Multiple Sclerosis Quality of Life-54 (MSQOL-54) [8] and the Health Utilities Index (HUI) [13], among others.

Previous research suggests that the HUI-III performs better than other health utility measures in discriminating between persons with MS with differing degrees of disability, being able to distinguish mild, moderate, severe, and very severely disabled persons [14]. It is unknown which instrument is most sensitive to the effect of comorbidity on HRQOL. This is relevant to future studies evaluating the impact of interventions directed at mitigating the effects of comorbidity on MS, and to studies aimed at evaluating the effects of comorbidity on MS. We aimed to compare the discriminative ability of HRQOL instruments, including the MSQOL-54, SF-36, SF-6D, and the HUI-III, in a prevalent MS population with and without comorbidity. We also examined whether the included measures performed similarly in persons with and without disability. Specifically, we hypothesized that: (1) generic measures would discriminate better between those with and without comorbidities than disease-specific measures; and (2) the HUI-III would discriminate best between persons with differing severities of disability.

## Methods

### Study population

The Alberta Multiple Sclerosis Initiative (TAMSI) was a longitudinal (July 2011–December 2013) observational study that used online questionnaires to collect patient-reported information about the safety, experiences, and outcomes following chronic cerebrospinal venous insufficiency (CCSVI) treatment. TAMSI had multiple modules and patients were able to select, based on their own schedules, which modules to complete. TAMSI was also designed to provide information about the health status and living conditions of all Albertans with MS, regardless of CCSVI treatment status.

For the current study, we included participants who completed all of the HRQOL measures (see below) and baseline surveys (demographics, MS characteristics, comorbidity status). To ensure the time period referred to in the questionnaires was consistent, the HRQOL surveys had to be completed within a two-week window. In addition, all baseline surveys had to be completed within 30 days of the HRQOL surveys. If participants filled out questionnaires at multiple time-points, the baseline information was used.

### Measures

Demographic information was collected, including age, sex, race, marital status, employment status, and level of education. Participants also reported characteristics of their MS including the date of onset of MS symptoms, use of disease modifying therapy, and disease course.

#### Disability

Disability status was reported by participants using the Patient-Determined Disease Steps (PDDS) [15], a validated self-report measure that strongly correlates with physician-assessed Expanded Disability Status Scale (EDSS) scores [16]. Based on the PDSS score [range 0 (no disability)-8 (bedbound)], disability was categorized into four groups: normal = 0; mild = 1–2; moderate = 3–5; and severe = 6–8.

#### HRQOL measures

Many measures have been used to quantify HRQOL in MS, including disease-specific and generic measures. Generic measures usually assess physical and mental health, are intended to be applied to any adult population, and are useful for making comparisons across diseases. Disease-specific measures are developed for a specific disease and may be more responsive to change in that specific disease than generic measures [17], but do not support comparisons across diseases, and may not capture the effects of comorbidities. Generic measures can be classified as health profiles or preference-based measures based on an individual preference or value for certain health states (utility measures). Health profile measures produce scores representing different domains of HRQOL, while utility measures result in a single weighted value that represents a person’s health state according to their preference. For this study we employed two HRQOL measures: a generic health utility measure, the HUI-III [18] and an MS-specific health profile measure, the MSQOL-54 [19]. From the MSQOL-54, we derived both the SF-36 [20], a generic health profile measure, and the SF-6D [21], a generic health utility measure. See Additional file 1: Table S1 for a comparison of the characteristics of all included HRQOL measures.

*Short Form-36*

The SF-36 is a 36-item generic measure of HRQOL that assesses eight domains: vitality, physical functioning, bodily pain, general health perceptions, physical role functioning, emotional role functioning, social role functioning, and mental health. The SF-36 generates two aggregate scores summarizing physical HRQOL (Physical Component Score, PCS-36) and mental HRQOL (Mental Component Score, MCS-36). Scores on the PCS-36 and MCS-36 range from 0 to 100, and are standardized to reflect a general population mean of 50 and standard deviation of 10. Higher scores indicate better HRQOL. The SF-36 has been extensively validated in MS, and has good reliability and validity [22, 23].

*SF-6D*

A health utility measure, the SF-6D is a subset of 11 questions from the SF-36, restructured into six dimensions: social functioning, physical functioning, mental health, pain, role limitations, and vitality. A single summary score is obtained, with possible scores ranging from 0.301 to 1.00, where 1.00 is perfect health. The validity and reliability of the SF-6D have been established in MS [24], and it has shown to distinguish between different levels of disability as measured by the EDSS [14].

*HUI-mark III*

The HUI-Mark III (HUI-III) is a 16-item generic multi-attribute utility-weighted measure of HRQOL that assesses eight domains, including hearing, speech, vision, ambulation, emotion, pain, cognition, and dexterity. An overall score of 1.00 on the HUI-III indicates perfect health, while a score of 0.00 is death, and negative scores (up to −0.36) represent health states worse than death. Previous research suggests the HUI-III outperforms other utility measures in MS [14, 24].

*MSQOL-54*

The MSQOL-54 is an MS-specific HRQOL measure that supplements the SF-36 with 18 disease-specific questions, measuring cognitive functioning, energy, pain, social functioning, sexual functioning, health anxiety, and overall quality of life. Similarly to the SF-36, two aggregate scores are generated, one for physical HRQOL (PCS-54) and one for mental HRQOL (MCS-54). The standardized scores range from 0 to 100, with higher scores indicating better HRQOL. The MSQOL-54 has been extensively validated, though the additional benefit of MS-specific items (over the SF-36) has been questioned [25, 26]. In this study, the sexual functioning subscale was optional, and participants were able to skip those questions if they felt they did not apply. For the present analyses, an alternate scoring system was used to account for the amount of missing items on the sexual functioning subscale (11.4% in this sample). A modified scoring algorithm was employed where the weight applied to the sexual functioning subscale was evenly distributed among the remaining seven subscales. This modified algorithm was applied to all participants, regardless if they had missing items on the sexual functioning subscale.

#### Comorbidity

Participants reported the presence or absence of comorbidities using a modified version of a validated questionnaire, with inclusion of additional comorbidities [27]. The comorbidities examined were: depression, anxiety disorders, bipolar disorder, alcoholism, hyperlipidemia, hypertension, migraine, lung trouble, thyroid disease, irritable bowel syndrome (IBS), osteoporosis, cataracts, diabetes, heart trouble, inflammatory bowel disease (IBD), rheumatoid arthritis, fibromyalgia, epilepsy, glaucoma, diseases of the arteries, lupus, and Parkinson’s disease. We analyzed comorbidities in four ways: (1) number of comorbidities (grouped as none, one, two, three or more); (2) presence/absence of each comorbidity; (3) any physical comorbidity (hyperlipidemia, hypertension, migraine, lung trouble, thyroid disease, IBS, osteoporosis, cataracts, diabetes, heart trouble, IBD, rheumatoid arthritis, fibromyalgia, epilepsy, glaucoma, diseases of the arteries, lupus, Parkinson’s disease); and (4) any psychiatric comorbidity (depression, anxiety disorders, bipolar disorder).

### Data analysis

Proportions and means (SD) were calculated for categorical and continuous data, respectively. Comorbidities were retained in the multivariate analyses if they had a prevalence of at least 5% in our sample (depression, hyperlipidemia, hypertension, migraine, lung trouble, thyroid disease, IBS, osteoporosis, anxiety disorders), to ensure enough individuals to provide a stable estimate.

#### HRQOL measure concordance

First, we examined the relationships between the HRQOL measures. We produced Spearman correlation matrices to compare correlations between each HRQOL measure. We considered correlations of 0.39 and below to be low, those between 0.40 and 0.59 as moderate, and those 0.60 and above as high [28].

#### HRQOL discriminant ability

Second, we examined the ability of each HRQOL measure to distinguish between groups. We did this for persons with different numbers of comorbidities (none, one, two, three or more), the absence/presence of each comorbidity, any physical comorbidity, any psychiatric comorbidity, and disability level (PDDS: normal, mild, moderate, severe). Comorbidity status or disability level were used as the intergroup factor in a one-way analysis of covariance (ANCOVA), with age, sex, disease course, and disability level (when not the intergroup factor) used as covariates. For all analyses the dependent variable was one of the HRQOL measures. Relative efficiency (RE) is a method of assessing the discriminative ability of different instruments to distinguish between groups; in this study we focused on groups according to comorbidity status or disability status. The RE of the instruments was calculated as the ratio of the between group ANCOVA F-statistics; Tukey statistics were used for the pairwise comparisons. [29] The instrument with the largest F-value/Tukey-value would have the greatest discriminative ability and was selected as the reference category (RE = 1) to determine the RE of all other instruments. Values of RE range from 0 to 1, where 1 indicates no difference in the instrument’s discriminative ability compared to the reference instrument. All reported *p*-values from the ANCOVA were corrected for multiple comparisons using Tukey’s procedure.

All data were analyzed using STATA version 11.0.

## Results

**Fig. 1:**
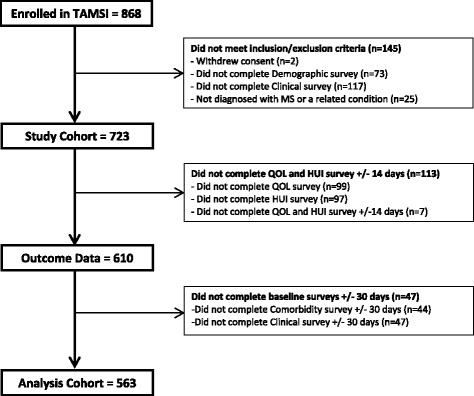
Participant flow diagram

Ultimately, 868 participants enrolled in this study, of whom 563 (64.9%) provided complete data for the HRQOL measures, disability, and comorbidity (Fig. 1) and were the subject of further analyses (Table 1). Their characteristics were similar to those expected for a prevalent MS population [30]. The distribution of comorbidities was as follows: none (*n* = 197, 35.0%); one (*n* = 163, 29.0%); two (*n* = 107, 19.0%), and three or more (*n* = 96, 17.1%) comorbidities. Physical comorbidities were present in 54.4% (*n* = 306) while psychiatric comorbidities were present in 29.1% (*n* = 164). The most common comorbidities were depression, hypertension, and hyperlipidemia (Table 2).

**Table 1:** Demographic and MS characteristics of TAMSI sample

Variable	Sample (*N* = 563)
Age (years), mean (SD)	47.6 (11.1)
Age of MS symptom onset (years), mean (SD)	33.5 (10.3)
Time since symptom onset (years), mean (SD)	14.3 (9.9)
Sex, N (%)	
Female	438 (77.8)
Male	125 (22.2)
Race, N (%)	
White	523 (96.1)
Non-White	21 (3.9)
Education, N (%)	
High School or Lower	149 (27.1)
College or Trade	212 (38.6)
University or Higher	188 (34.2)
Marital Status, N (%)	
Single	71 (12.8)
Married/Common Law	415 (75.0)
Widowed/Divorced/Separated	67 (12.1)
Employment Status, N (%)	
Employed	266 (48.2)
Unemployed	286 (51.8)
Geographic Area, N (%)	
Calgary	237 (42.1)
Edmonton	176 (31.3)
Central Alberta	83 (14.7)
Northern Alberta	39 (6.9)
Southern Alberta	28 (5.0)
Family History of MS, N (%)	
Yes	160 (30.0)
No	373 (70.0)
Definite MS, N (%)	
Yes	525 (93.3)
No	8 (6.7)
Disease Course, N (%)^a^	
RRMS	363 (69.1)
SPMS	91 (17.3)
PPMS	50 (9.5)
Uncertain	21 (4.0)
Disability Status (PDDS)	
Normal	143 (25.4)
Mild	127 (22.6)
Moderate	182 (32.3)
Severe	111 (19.7)
DMT Use, N (%)	
Never	178 (32.1)
Past	95 (17.1)
Current	281 (50.7)

*PDDS* Patient Determined Disease Steps

*DMT* Disease-modifying therapy

^a^Note this category represents the 525 persons who indicated having definite MS

**Table 2:** Comorbidities in TAMSI sample

Comorbidity	N (%)
Depression	150 (26.6)
Hypertension	86 (15.3)
Hyperlipidemia	85 (15.1)
Migraine	84 (14.9)
Lung Trouble	49 (8.7)
Thyroid Disease	44 (7.8)
Irritable Bowel Syndrome (IBS)	42 (7.5)
Osteoporosis	42 (7.5)
Anxiety Disorders	38 (6.7)
Cataracts	24 (4.3)
Diabetes	20 (3.6)
Heart Trouble	14 (2.5)
Inflammatory Bowel Disease (IBD)	11 (2.0)
Rheumatoid Arthritis	11 (2.0)
Fibromyalgia	9 (1.6)
Epilepsy	7 (1.2)
Bipolar Disorder	6 (1.1)
Glaucoma	5 (0.9)
Diseases of the Arteries	4 (0.7)
Lupus	4 (0.7)
Alcoholism	3 (0.5)
Parkinson’s Disease	0 (0.0)

The mean (SD) score was 38.4 (12.3) for the PCS-36, 48.5 (11.9) for the MCS-36, 0.56 (0.32) for the HUI-III, 0.68 (0.13) for the SF-6D, 57.4 (22.2) for the PCS-54 and 63.2 (19.1) for the MCS-54.

### Comorbidity

**Fig. 2:**
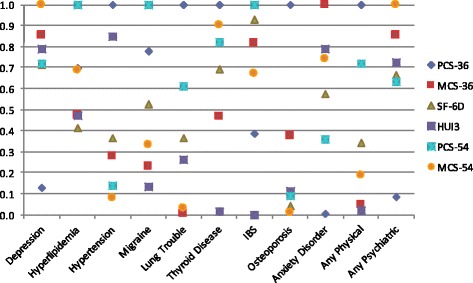
Relative efficiency of HRQOL measures to discriminate absence/presence of comorbidities

Mean HRQOL scores for all scales were highest in those without comorbidities, and decreased with an increasing number of comorbidities (Additional file 2: Figure S1). The ANCOVA revealed statistically significant overall effects of the number of comorbidities for all HRQOL measures (PCS-36 F = 10.70, *p* < 0.0001; MCS-36 F = 9.28, *p* < 0.0001; SF-6D F = 11.56, *p* < 0.0001, HUI-III F = 9.68, *p* < 0.0001; PCS-54 F = 19.05, *p* < 0.0001; MCS-54 F = 13.47, *p* < 0.0001; Additional file 3: Table S2), indicating that each measure was able to distinguish between people with differing numbers of comorbidities (none, one, two, three or more). Further pairwise comparisons showed that no measure was able to distinguish between persons without comorbidities and persons with one comorbidity or persons with two comorbidities versus three or more. The PCS-36, SF-6D, HUI-III, and PCS-54 were able to distinguish between all other combinations of the number of comorbidities (all *p* < 0.05), while the MCS-54 was able to distinguish between persons with none or two, none or three or more, and one or three or more comorbidities; and the MCS-36 was only able to distinguish between persons with none or three or more comorbidities. The RE of each instrument by comorbidity status is displayed in Fig. 2.

All HRQOL measures were able to distinguish between those with and without psychiatric comorbidities (all *p* < 0.005). Neither the MCS measures, nor the HUI-III, were able to distinguish between those with and without physical comorbidities. When each comorbidity was examined individually, all HRQOL measures were able to distinguish between those persons with or without depression (all *p* < 0.001) (Additional file 3: Table S2). Hyperlipidemia was distinguishable by the PCS-36, PCS-54, and MCS-54; migraine by the PCS-36, SF-6D, and PCS-54; lung trouble by the PCS-36; and anxiety disorders by all except the PCS-36. None of the HRQOL measures were able to distinguish participants with or without hypertension, thyroid disease, IBS, or osteoporosis. Based on the F-test, the PCS-54 performed the best for number of comorbidities, while the PCS-36 performed best for any physical comorbidity and the MCS-54 performed best for any psychiatric comorbidity.

### Disability

Scores on the HRQOL measures decreased with increasing disability on the PDDS, except for the MCS-36, where mean scores were similar across the disability levels (data not shown).

**Fig. 3:**
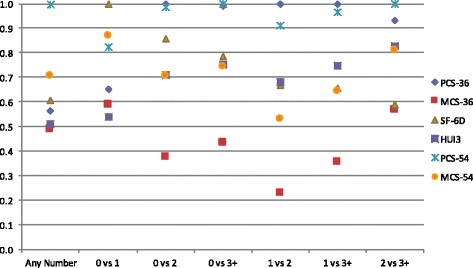
Relative efficiency of HRQOL measures to discriminate number of comorbidities

All HRQOL measures were able to distinguish between disability groups (PCS-36 F = 132.53, *p* < 0.0001; MCS-36 F = 3.84, *p* = 0.01; SF-6D F = 45.18, *p* < 0.0001; HUI-III F = 72.27, *p* < 0.0001; PCS-54 F = 83.58, *p* < 0.0001; MCS-54 F = 32.12, *p* < 0.0001). Pairwise comparisons indicated that the PCS-36, SF-6D, HUI-III, and PCS-54 were able to distinguish between all disability groups (Table 3). The MCS-54 was able to distinguish between all groups except persons with mild versus moderate disability. The MCS-36 was only able to distinguish between persons with normal versus mild disability, and persons with mild versus moderate disability. Based on F-tests, the PCS-36 performed best overall; however the HUI-III was better able to distinguish between individuals with moderate versus severe disability. The RE of each instrument by disability level is displayed in Fig. 3.

**Table 3:** Analysis of covariance for HRQOL measures by disability level*

Dependent Variable	PCS-36		MCS-36		SF-6D		HUI-III		PCS-54		MCS-54	
Independent Factor Variable	F-test	p-value	F-test	p-value	F-test	p-value	F-test	p-value	F-test	p-value	F-test	p-value
Disability Status	132.53	**<0.0001**	3.84	**0.01**	45.18	**<0.0001**	72.27	**<0.0001**	83.58	**<0.0001**	32.12	**<0.0001**
Normal vs. Mild	12.68	**<0.05**	4.03	**<0.05**	9.94	**<0.05**	8.80	**<0.05**	11.98	**<0.05**	8.15	**<0.05**
Normal vs. Moderate	26.72	**<0.05**	0.30	NS	13.69	**<0.05**	15.68	**<0.05**	19.71	**<0.05**	10.82	**<0.05**
Normal vs. Severe	34.65	**<0.05**	0.67	NS	20.58	**<0.05**	26.93	**<0.05**	26.47	**<0.05**	16.98	**<0.05**
Mild vs. Moderate	14.04	**<0.05**	4.33	**<0.05**	3.75	**<0.05**	6.88	**<0.05**	7.73	**<0.05**	2.67	NS
Mild vs. Severe	21.96	**<0.05**	3.36	NS	10.64	**<0.05**	18.13	**<0.05**	14.48	**<0.05**	8.83	**<0.05**
Moderate vs. Severe	7.93	**<0.05**	0.97	NS	6.90	**<0.05**	11.25	**<0.05**	6.75	**<0.05**	6.16	**<0.05**

*Adjusted for age (continuous), sex, and disease course; **BOLD** type indicates *p* < 0.05; *NS* not significant

## Discussion

In this study, we evaluated the discriminative ability of multiple HRQOL measures to distinguish between persons with and without comorbidities in MS. We also examined the ability of these HRQOL scales to discriminate between varying levels of patient self-reported disability. The examined scales were only able to distinguish between persons with and without some comorbidities, particularly depression, and differed in their ability to distinguish individuals at higher levels of disability. The MCS-36 was not a good tool to distinguish between disability levels or number of comorbidities. The PCS-54 was slightly better than the generic tools at distinguishing between differing numbers of comorbidities, but it was comparable to the generic tools for levels of disability. In general, the MCS measures performed worse than the PCS measures for both the SF-36 and MSQOL-54, with the MCS-54 outperforming the MCS-36 for both comorbidity and disability discrimination. Of the utility measures, the SF-6D and HUI-III performed comparably for discriminating comorbidity and disability statuses.

In our study no measure was able to distinguish between those with no comorbidities and those with only one comorbidity, but the PCS-54 was very good at distinguishing between persons with different numbers of comorbidities, and the PCS-36, MCS-36, SF-6D, HUI-III, and MCS-54 were also adequate. All HRQOL scales were very good at distinguishing persons with depression. Some HRQOL scales were also good at distinguishing those with hyperlipidemia and anxiety disorders. Overall the PCS-54 was slightly better at distinguishing between differing numbers of comorbidities than the generic tools. Of the utility measures, the SF-6D and HUI-III performed comparably for discriminating comorbidity statuses, although the SF-6D distinguished between persons with and without any psychiatric comorbidity and between persons with and without migraine; whereas the HUI-III did not. It is possible to achieve more precision with the PCS-54 compared with the PCS-36, SF-6D or the HUI-III but this comes at the cost of additional questions (54 vs. 36, 11, 16). However, the length of the HRQOL tool does not appear to affect a subject’s perception of the tool’s utility [26]. Prior work has not identified clear differences in reliability and validity between disease-specific and general instruments when used cross-sectionally [26].

HRQOL scores were generally lowest in those persons with severe disability in the present study consistent with previous work [14]. There was a dose-response relationship between disability level and scores on all HRQOL measures, except the MSC-36. A prior study examining the utility of generic HRQOL measures across varying levels of MS-disability found that the HUI-III performed best to discriminate between all levels of disability [14]. As correlations between the MCS-54 and MCS-36 were very high, there may not be additional value in the extra 18 items on the MS-specific questionnaire (especially considering the amount of missing items on the optional sexual functioning subscale). We used a modified MSQOL-54 scoring algorithm to account for a large amount of missing data on the sexual function subscale. Missing data on this subscale is not uncommon in MS research [26], and other studies have employed modified algorithms to account for this issue [31]. We found the HUI-III was best able to discriminate between higher levels of disability (moderate and severe) as compared to the other generic and disease-specific measures. This is especially relevant in the context of progressive MS research and trials, where appropriate patient reported outcome measures must be carefully selected to ensure adequate sensitivity to change [32].

We examined generic and disease-specific health profile and utility measures of HRQOL in a large, well-described sample of persons with MS [33]. We explored the use of these tools across disability levels and comorbidity status, which has not been previously reported in the MS population. All tools except the SF-36 and MSQOL-54 are utility scales designed to put the effects of diverse illnesses onto a single scale; this is valuable when assessing patients with comorbidities. Limitations of the present study include the online nature of the survey, which may bias toward the inclusion of participants who are more educated, and with a higher income [33]. As noted previously, participants in this study were more likely to have a university education and to live in a neighborhood with a higher median income, compared to a reference MS population. The administration of the scales was not entirely independent, though the SF-36 was designed to be administered in this manner. This study was cross-sectional and therefore we did not examine the use of these tools over time; future studies should explore the responsiveness of these tools to change and individual response shift. Due to the online nature of the survey it was not possible for respondents to skip questions; as such scales were completed in their entirety or not at all, which may have resulted in more non-response on the HRQOL questionnaires. Whereas this study provided a broad-based comparison of two types of instruments, future studies seeking to compare specific instruments should consider adopting a priori criteria for assessing instrument performance.

## Conclusions

Our findings suggest that future studies aimed at evaluating the effects of comorbidity or of comorbidity treatment on HRQOL in MS, may reasonably choose to employ generic or disease-specific measures depending on the MS population of interest. Disease-specific measures would discriminate better between those with and without comorbidities, and between those with differing numbers of comorbidities than generic measures. In contrast, the HUI-III would discriminate best between persons with moderate and severe disability. In studies in which the participants enrolled may have high levels of disability, the HUI-III appears best able to discriminate differences between participants, suggesting this may be a good measure for trials in progressive MS, although further evaluation is needed.

## Additional files

Additional file 1: **Table S1**. Comparison of Health Related Quality of Life Measures. (DOCX 12 kb)

Additional file 2: **Figure S1**. Mean score on HRQOL measures according to the number of comorbidities. (DOCX 29 kb)

Additional file 3: **Table S2**. Analysis of covariance for HRQOL measures by comorbidity status. (DOCX 14 kb)

## Abbreviations

CCSVI: Chronic cerebrospinal venous insufficiency
EDSS: Expanded Disability Status Scale
HRQOL: Health Related Quality of Life
HUI: Health Utilities Index
IBD: Inflammatory bowel disease
IBS: Irritable bowel syndrome
MCS: Mental component score
MS: Multiple Sclerosis
MSQOL-54: Multiple Sclerosis Quality of Life-54
PCS: Physical component score
PDDS: Patient-Determined Disease Steps
RE: Relative efficiency
SF: Short Form
TAMSI: The Alberta Multiple Sclerosis Initiative
