# Supplementary material for: Discriminative ability of quality of life measures in multiple sclerosis
Source: Health Qual Life Outcomes. 2017 Dec 21;15:246. doi: 10.1186/s12955-017-0828-0 (PMC5740906; doi:10.1186/s12955-017-0828-0)
Supplement: Supplementary file 1 — Table S1. Comparison of Health Related Quality of Life Measures. (DOCX 12 kb) [file 12955_2017_828_MOESM1_ESM.docx]

**Table S1**. Comparison of Health Related Quality of Life Measures

|  | **Quality of Life Measures** | | **Utility Measures** | |
| --- | --- | --- | --- | --- |
|  | *SF-36* | *MSQOL-54* | *SF-6D* | *HUI-III* |
| Number of Items | 36 | 54 | 11 | 16 |
| Range of Scores | 0 – 100 | 0 – 100 | 0.301 – 1.00 | -0.36 – 1.00 |
| Generic/Disease-Specific | Generic | Disease-Specific | Generic | Generic |
| Physical Subscale | ✓ | ✓ |  |  |
| Mental Subscale | ✓ | ✓ |  |  |
| Domains |  |  |  |  |
| Vitality (energy) | ✓ | ✓ | ✓ |  |
| Physical Functioning | ✓ |  |  |  |
| Bodily Pain | ✓ | ✓ | ✓ | ✓ |
| General Health Perceptions | ✓ |  |  |  |
| Physical Role Functioning | ✓ |  | ✓ |  |
| Emotional Role Functioning | ✓ |  |  |  |
| Social Role Functioning | ✓ | ✓ | ✓ |  |
| Mental Health | ✓ |  | ✓ |  |
| Sexual Functioning |  | ✓ |  |  |
| Health Anxiety |  | ✓ |  |  |
| Overall Quality of Life |  | ✓ |  |  |
| Role Limitations |  |  | ✓ |  |
| Hearing |  |  |  | ✓ |
| Speech |  |  |  | ✓ |
| Vision |  |  |  | ✓ |
| Ambulation |  |  |  | ✓ |
| Emotion |  |  |  | ✓ |
| Cognition |  | ✓ |  | ✓ |
| Dexterity |  |  |  | ✓ |
