# Supplementary material for: Discriminative ability of quality of life measures in multiple sclerosis
Source: Health Qual Life Outcomes. 2017 Dec 21;15:246. doi: 10.1186/s12955-017-0828-0 (PMC5740906; doi:10.1186/s12955-017-0828-0)
Supplement: Supplementary file 2 — Figure S1. Mean score on HRQOL measures according to the number of comorbidities. (DOCX 29 kb) [file 12955_2017_828_MOESM2_ESM.docx]

**Figure S1**. Mean score on HRQOL measures according to the number of comorbidities

Note: SF-6D and HUI-III mean scores and standard deviations were multiplied by 100 to aid in comparison
