# Supplementary material for: Discriminative ability of quality of life measures in multiple sclerosis
Source: Health Qual Life Outcomes. 2017 Dec 21;15:246. doi: 10.1186/s12955-017-0828-0 (PMC5740906; doi:10.1186/s12955-017-0828-0)
Supplement: Supplementary file 3 — Table S2. Analysis of covariance for HRQOL measures by comorbidity status. (DOCX 14 kb) [file 12955_2017_828_MOESM3_ESM.docx]

**Table S2**. Analysis of covariance for HRQOL measures by comorbidity status

| **Dependent Variable** | **PCS-36** | | **MCS-36** | | **SF-6D** | | **HUI-III** | | **PCS-54** | | **MCS-54** | |
| --- | --- | --- | --- | --- | --- | --- | --- | --- | --- | --- | --- | --- |
| **Independent Factor Variable** | **F-test** | **p-value** | **F-test** | **p-value** | **F-test** | **p-value** | **F-test** | **p-value** | **F-test** | **p-value** | **F-test** | **p-value** |
| Number of Comorbidities | 10.70 | **<0.0001** | 9.28 | **<0.0001** | 11.56 | **<0.0001** | 9.68 | **<0.0001** | 19.05 | **<0.0001** | 13.47 | **<0.0001** |
| Any Physical Comorbidity | 12.30 | **0.0005** | 0.58 | 0.45 | 4.19 | **0.04** | 0.23 | 0.63 | 8.84 | **0.003** | 2.30 | 0.13 |
| Any Psychiatric Comorbidity | 8.58 | **0.004** | 85.23 | **<0.0001** | 66.32 | **<0.0001** | 72.10 | **<0.0001** | 63.10 | **<0.0001** | 100.02 | **<0.0001** |
| None vs. One | 1.84 | NS | 1.66 | NS | 2.83 | NS | 1.52 | NS | 2.33 | NS | 2.46 | NS |
| None vs. Two | 8.61 | **<0.05** | 3.23 | NS | 7.36 | **<0.05** | 6.12 | **<0.05** | 8.50 | **<0.05** | 6.06 | **<0.05** |
| None vs. Three or more | 11.85 | **<0.05** | 5.20 | **<0.05** | 9.41 | **<0.05** | 8.99 | **<0.05** | 11.97 | **<0.05** | 8.88 | **<0.05** |
| One vs. Two | 6.77 | **<0.05** | 1.56 | NS | 4.53 | **<0.05** | 4.60 | **<0.05** | 6.17 | **<0.05** | 3.60 | NS |
| One vs. Three or more | 10.01 | **<0.05** | 3.54 | NS | 6.58 | **<0.05** | 7.47 | **<0.05** | 9.65 | **<0.05** | 6.42 | **<0.05** |
| Two vs. Three or more | 3.24 | NS | 1.98 | NS | 2.05 | NS | 2.87 | NS | 3.48 | NS | 2.82 | NS |
| Depression | 12.04 | **0.0006** | 80.81 | **<0.0001** | 67.67 | **<0.0001** | 75.04 | **<0.0001** | 68.20 | **<0.0001** | 95.09 | **<0.0001** |
| Hyperlipidemia | 5.61 | **0.02** | 3.80 | 0.05 | 3.31 | 0.07 | 3.80 | 0.05 | 8.03 | **0.005** | 5.52 | **0.02** |
| Hypertension | 1.30 | 0.26 | 0.36 | 0.55 | 0.47 | 0.49 | 1.10 | 0.30 | 0.18 | 0.67 | 0.10 | 0.75 |
| Migraine | 6.02 | **0.01** | 1.80 | 0.18 | 4.06 | **0.04** | 1.03 | 0.31 | 7.76 | **0.006** | 2.57 | 0.11 |
| Lung Trouble | 6.41 | **0.01** | 0.03 | 0.87 | 2.33 | 0.13 | 1.69 | 0.19 | 3.91 | 0.05 | 0.19 | 0.67 |
| Thyroid Disease | 1.89 | 0.17 | 0.88 | 0.35 | 1.31 | 0.25 | 0.03 | 0.86 | 1.55 | 0.21 | 1.70 | 0.19 |
| Irritable Bowel Syndrome | 1.29 | 0.26 | 2.75 | 0.10 | 3.13 | 0.08 | 0.00 | 0.97 | 3.37 | 0.07 | 2.25 | 0.13 |
| Osteoporosis | 1.01 | 0.31 | 0.38 | 0.54 | 0.04 | 0.85 | 0.11 | 0.74 | 0.09 | 0.76 | 0.01 | 0.92 |
| Anxiety Disorders | 0.03 | 0.86 | 19.28 | **<0.0001** | 11.08 | **0.0009** | 15.21 | **<0.0001** | 6.91 | **0.01** | 14.31 | **0.0002** |

*Note: Adjusted for age (continuous), sex, disease course, and disability; **BOLD** type indicates p<0.05; NS=not significant
